# Drugs that target early stages of *Onchocerca volvulus*: A revisited means to facilitate the elimination goals for onchocerciasis

**DOI:** 10.1371/journal.pntd.0009064

**Published:** 2021-02-18

**Authors:** Shabnam Jawahar, Nancy Tricoche, Christina A. Bulman, Judy Sakanari, Sara Lustigman

**Affiliations:** 1 Molecular Parasitology, Lindsey F. Kimball Research Institute, New York Blood Center, New York, New York, United States of America; 2 Department of Pharmaceutical Chemistry, University of California San Francisco, San Francisco, California, United States of America; University of Zurich, SWITZERLAND

## Abstract

Several issues have been identified with the current programs for the elimination of onchocerciasis that target only transmission by using mass drug administration (MDA) of the drug ivermectin. Alternative and/or complementary treatment regimens as part of a more comprehensive strategy to eliminate onchocerciasis are needed. We posit that the addition of “prophylactic” drugs or therapeutic drugs that can be utilized in a prophylactic strategy to the toolbox of present microfilaricidal drugs and/or future macrofilaricidal treatment regimens will not only improve the chances of meeting the elimination goals but may hasten the time to elimination and also will support achieving a sustained elimination of onchocerciasis. These “prophylactic” drugs will target the infective third- (L3) and fourth-stage (L4) larvae of *Onchocerca volvulus* and consequently prevent the establishment of new infections not only in uninfected individuals but also in already infected individuals and thus reduce the overall adult worm burden and transmission. Importantly, an effective prophylactic treatment regimen can utilize drugs that are already part of the onchocerciasis elimination program (ivermectin), those being considered for MDA (moxidectin), and/or the potential macrofilaricidal drugs (oxfendazole and emodepside) currently under clinical development. Prophylaxis of onchocerciasis is not a new concept. We present new data showing that these drugs can inhibit L3 molting and/or inhibit motility of L4 at IC_50_ and IC_90_ that are covered by the concentration of these drugs in plasma based on the corresponding pharmacological profiles obtained in human clinical trials when these drugs were tested using various doses for the therapeutic treatments of various helminth infections.

*Onchocerca volvulus* is an obligate human parasite and the causative agent for onchocerciasis, which is a chronic neglected tropical disease prevalent mostly in the sub-Saharan Africa. In 2017, 20.9 million people were infected, with 14.6 million having skin pathologies and 1.15 million having vision loss [1]. The socioeconomic impact of onchocerciasis and the debilitating morbidity caused by the disease prompted the World Health Organization (WHO) to initiate control programs that were first focused on reducing onchocerciasis as a public health problem, and since 2012, the ultimate goal is to eliminate it by 2030 [2]. Over the years, WHO sponsored and coordinated 3 major programs: The Onchocerciasis Control Programme (OCP), the African Programme for Onchocerciasis Control (APOC), and the Onchocerciasis Elimination Program of the Americas (OEPA). Since 1989, the control measures depended on mass drug administration (MDA) annually or biannually with ivermectin, which targets the transmitting stage of parasite, the microfilariae [3–5]. However, several issues have been identified with the current MDA programs including the need to expand the treatment to more populations depending on baseline endemicity and transmission rates [2,6]. Moreover, it became apparent that alternative and/or complementary treatment regimens as part of a more comprehensive strategy to eliminate onchocerciasis are needed [2]. Ivermectin has only mild to moderate effects on the adult stages of the parasite [7–9], and there are communities in Africa where the effects of ivermectin are suboptimal [10]. It is also contraindicated in areas of *Loa loa* co-endemicity [11], as well as in children under the age of 5 and in pregnant women. By relying only on MDA with ivermectin, the most optimistic mathematical modeling predicts that elimination will occur only in 2045 [12].

To support the elimination agenda, much of the recent focus has been on improving efficacy outcomes through improved microfilariae control with moxidectin and the discovery of macrofilaricidal drugs that target the adult *O*. *volvulus* parasites [13–18]. We posit that the addition of “prophylactic” drugs or therapeutic drugs that can be utilized in a prophylactic strategy to the toolbox of present microfilaricidal drugs and/or future macrofilaricidal treatment regimens will not only improve the chances of meeting the elimination goals but may also hasten the time for elimination and support achieving a sustained elimination of onchocerciasis. These “prophylactic” drugs will target the infective third- (L3) and fourth-stage (L4) larvae of *O*. *volvulus* and consequently prevent the establishment of new infections not only in the uninfected individuals but also in the already infected individuals and thus reduce the overall adult worm burden and transmission. Importantly, an effective prophylactic treatment regimen can utilize drugs that are already part of the onchocerciasis elimination program (ivermectin), those being considered for MDA (moxidectin) [19,20], and/or the potential macrofilaricidal drugs (oxfendazole and emodepside) currently under clinical development [21].

Prophylaxis of onchocerciasis is not a new concept. In the 1980s, once ivermectin was introduced as a “prophylactic” drug against the filarial dog heartworm, *Dirofilaria immitis* [22], its prophylactic effects were also examined in *Onchocerca* spp. In chimpanzees, a single dose of ivermectin (200 μg/kg) was highly protective (83% reduction in patent infections) when given at the time of the experimental infection and tracked for development of patency over 30 months. It was, however, much less effective (33% reduction in patent infections) when given 1 month postinfection with the L3s, at which time the L4s had already developed [23]. Moreover, monthly treatment with ivermectin at either 200 μg/kg or 500 μg/kg for 21 months completely protected naïve calves against the development of *O*. *ochengi* infection as compared to untreated controls, which were 83% positive for nodules and 100% positive for patency [24]. When naïve calves exposed to natural infection were treated with either ivermectin (150 μg/kg) or with moxidectin (200 μg/kg) monthly or quarterly, none of the animals developed detectable infections after 22 months of exposure, except 2 animals in the quarterly ivermectin treated group which had 1 nodule each; in the non-treated control group, the nodule prevalence was 78.6% [25]. These prophylactic studies in calves exposed to natural infections clearly demonstrated that monthly or quarterly treatments with ivermectin and/or moxidectin over 22 months were highly efficacious against the development of new infections. When ivermectin was administered in a highly endemic region of onchocerciasis in Cameroon every 3 months over a 4-year period, it resulted in reduced numbers of new nodules (17.7%) when compared to individuals who were treated annually. This recent study suggests that ivermectin may have also a better prophylactic effect in humans when administered quarterly [26].

Importantly, moxidectin, a member of the macrocyclic lactone family of anthelmintic drugs, also used in veterinary medicine like ivermectin [20], was recently approved for the treatment of onchocerciasis as a microfilaricidal drug in individuals over the age of 12 [20]. In humans, a single dose of moxidectin (8 mg) appeared to be more efficacious than a single dose of ivermectin (150 μg/kg) in terms of lowering microfilarial loads [17]. Modeling has shown that an annual treatment with moxidectin and a biannual treatment with ivermectin would achieve similar reductions in the duration of the MDA programs when compared to an annual treatment with ivermectin [27].

In our efforts to identify macrofilaricidal drugs, we tested a selection of drugs for their ability to inhibit the molting of *O*. *volvulus* L3 to L4 as part of the in vitro drug screening funnel [13,28–31]. With some being highly effective, we decided to also examine the effects of the known MDA drugs and those already in clinical development for macrofilaricidal effects on molting of L3 and the motility of L4 (S1 Text) as potential “prophylactic” drugs. When ivermectin and moxidectin were evaluated, we found that both drugs were highly effective as inhibitors of molting: IC_50_ of 1.048 μM [918.86 ng/ml] and IC_90_ of 3.73 μM [2,949.1 ng/ml] for ivermectin and IC_50_ of 0.654 μM [418.43 ng/ml] and IC_90_ of 1.535 μM [985.3 ng/ml] for moxidectin (Table 1 and S1 Fig), with moxidectin being more effective than ivermectin. When both drugs were tested against the L4, we found that both drugs inhibited the motility of L4s after 6 days of treatment: Ivermectin had an IC_50_ of 1.38 μM [1,207.6 ng/ml] and IC_90_ of 31.45 μM [27,521.9 ng/ml] (Table 1 and S1 Fig), while moxidectin had an IC_50_ of 1.039 μM [665.4 ng/ml] and IC_90_ of approximately 30 μM [approximately 19,194 ng/ml] (Table 1 and S1 Fig). Interestingly, when the treatment of L4 with both drugs was prolonged, the IC_50_ values for the inhibition of L4 motility on day 11 with ivermectin and moxidectin were 0.444 μM and 0.380 μM, respectively. Significantly, from the prospect of employing both drugs for prophylaxis against new infections with *O*. *volvulus*, moxidectin (8 mg) has an advantage as it achieves a maximum plasma concentration of 77.2 ± 17.8 ng/ml, is metabolized minimally, and has a half-life time of 40.9 ± 18.25 days with an area under the curve (AUC) of 4,717 ± 1,494 ng*h/ml in healthy individuals [32], which covers the experimental IC_50_ achieved by moxidectin for inhibiting both L3 molting and L4 motility, and the IC_90_ for L3s. In comparison, ivermectin reaches a maximum plasma concentration of 54.4 ± 12.2 ng/ml with a half-life of 1.5 ± 0.43 days and an AUC of 3,180 ± 1,390 ng*h/ml in healthy humans [33], which only covers the IC_50_ for inhibiting molting of L3 and motility of L4. We therefore reason that based on the significantly improved pharmacokinetic profile of moxidectin and its efficacy against both L3 and L4 larvae in vitro (Table 1), it might have a better “prophylactic” profile than ivermectin for its potential to interrupt the development of new *O*. *volvulus* infections, and thus ultimately affect transmission and further support the elimination of onchocerciasis. Adding to moxidectin’s significance, in dogs, it is a highly effective prophylactic drug against ivermectin-resistant *D*. *immitis* strains [19], an important attribute in the event that a suboptimal responsiveness to ivermectin treatment becomes more widespread in the onchocerciasis endemic regions of Africa. Testing the potential effect of moxidectin on the viability or development of transmitted L3 larvae was already recommended by Awadzi and colleagues in 2014 [34], when the excellent half-life of moxidectin in patients with onchocerciasis was realized. We have to acknowledge, however, that the key parameters that can predict the potency of a drug is actually a combination of exposure (drug concentrations) at the site of action and the duration of that exposure that is above the determined IC_50_/IC_90_. As we have access to only the AUC, half-life, and Cmax data for each of the in vitro–tested drugs, the use of plasma concentrations for predicting the anticipated potency of these putative “prophylactic” drugs in vivo has to be further assessed with care during clinical trials.

**Table 1:** Inhibition of *O*. *volvulus* L3 molting and L4 motility in vitro by the prospective prophylactic drugs and their essential pharmacokinetic parameters at doses currently used or deemed safe for use in humans.

	Drug	Ivermectin		Moxidectin		Albendazole		Oxfendazole		Emodepside	
						Albendazole sulfoxide					
		IC_50_ μM (conc in ng/ml)	IC_90_ μM (conc in ng/ml)	IC_50_ μM (conc in ng/ml)	IC_90_ μM (conc in ng/ml)	IC_50_ μM (conc in ng/ml)	IC_90_ μM (conc in ng/ml)	IC_50_ μM (conc in ng/ml)	IC_90_ μM (conc in ng/ml)	IC_50_ μM (conc in ng/ml)	IC_90_ μM (conc in ng/ml)
In vitro drug testing with *O*. *volvulus* larvae	**Inhibition of L3 molting^a^**	1.048 (918.86 ng/ml)	3.730 (2,949.1 ng/ml)	0.654 (418.43 ng/ml)	1.535 (985.3 ng/ml)	0.007 (1.9 ng/ml)	0.023 (5.8 ng/ml)	0.034 (10.7 ng/ml)	0.071 (22.4 ng/ml)	0.0007 (0.8 ng/ml)	0.002 (2.2 ng/ml)
						0.008 (2.25 ng/ml)	0.07 (19.69 ng/ml)				
	**Inhibition of L4 motility^b^**	1.38 (1,207 ng/ml)	31.45 (27,521 ng/ml)	1.039 (665 ng/ml)	approximately 30 (approximately 19,194 ng/ml)	>2 μM				0.0005 (0.6 ng/ml)	0.078 (87.3 ng/ml)
Pharmacokinetic profiles extracted from data collected during clinical trials in humans^c^	**Dose**	150 μg/kg		8 mg		400 mg		15 mg/kg	30 mg/kg	1 mg	40 mg
	**Cmax (plasma) ng/ml**	54.4 ± 12.2		77.2 ± 17.8		24.5	288^d^	6,250 ± 1,390	5,300 ± 1,690	18.6	434
	**Half-life t1/2 (h)**	36.6 ± 10.2		981 ± 438		1.53	8.56^d^	9.97 ± 2.22	9.82 ± 3.46	42.7	392
	AUC (ng*h/ml)	3,180 ± 1,390		4,717 ± 1,494		73	3,418^d^	99,500 ± 2,440	78,300 ± 2,830	100	3,320
	**Citations**	[33]		[32]^e^		[41]		[42]		[43]	

^a^*O*. *volvulus* L3 obtained from infected *Simulium* sp. were washed and distributed at *n* = approximately 10 larvae per well and cocultured in contact with naïve human peripheral blood mononuclear cells for a period of 6 days with or without the respective drugs in vitro (S1 Text) and as previously described [13,30]. Ivermectin (PHR1380, Sigma-Aldrich, St. Louis, Missouri, United States of America) and moxidectin (PHR1827, Sigma-Aldrich) were tested in the range of 0.01–10 μM; albendazole (A4673, Sigma-Aldrich), albendazole sulfoxide (35395, Sigma-Aldrich), and oxfendazole (31476, Sigma-Aldrich) in the range of 1–3 μM; and emodepside (Bayer) in the range of 0.3–1 μM using 3-fold dilutions. On day 6, molting of L3 worms was recorded. Each condition was tested in duplicate and repeated at least once. The IC_50_ and IC_90_ were derived from nonlinear regression (curve fit) analysis on GraphPad Prism 6 with 95% confidence intervals.

^b^L3s were allowed to molt to L4 in the presence of PBMCs and on day 6 when molting was complete the L4 larvae were collected and distributed at 6–8 worms per well and treated with the respective concentrations of drugs [ivermectin and moxidectin: 0.01–30 μM at 3-fold dilutions and emodepside: 0.03–3 μM at 10-fold dilutions and 10 μM] for a period of 6 days. Inhibition of *O*. *volvulus* L4 motility was recorded as described [13,30]; representative videos of motility and inhibited motility can be viewed in Voronin and colleagues [30], S1–S3 Videos. Each condition was tested in duplicate and repeated at least once. The IC_50_ and IC_90_ were derived from nonlinear regression (curve fit) analysis on GraphPad Prism 6 with 95% confidence intervals.

^c^Information regarding the pharmacokinetic profiles of each drug was extracted from public data collected during the corresponding clinical trial(s) in humans, which are also referenced.

^d^Pharmacokinetic parameters of albendazole sulfoxide, the predominant metabolite of albendazole.

^e^Additional pharmacokinetics parameters for moxidectin not only in heathy individual but also in those living in Africa can be found on the moxidectin FDA prescribing information website: https://www.drugs.com/pro/moxidectin.html. In patients with onchocerciasis, it is reported that a single dose of moxidectin (8 mg) achieves a maximum plasma concentration of 63.1 ± 20.0 ng/ml, and it has a half-life time of 559 ± 525 days with an AUC of 2,738 ± 1,606 ng*h/ml.

AUC, area under the curve; Cmax, maximum plasma concentration.

The prospects for identifying additional “prophylactic” drugs against *O*. *volvulus* increased when we tested 3 other drugs: albendazole, already in use for controlling helminth infections in humans; and oxfendazole and emodepside, being tested by the Drugs for Neglected Diseases initiative (DNDi) as potential repurposed macrofilaricidal drugs for human indications [21]. Albendazole is a primary drug of choice for MDA treatment of soil-transmitted helminths (STH; hookworms, whipworms [in combination with oxantel pamoate], and ascarids) [35], as well as for the elimination of lymphatic filariasis in Africa when used in combination with ivermectin [36]. Oxfendazole, a member of the benzimidazole family, is currently indicated for the treatment of a range of lung and gastrointestinal parasites in cattle and other veterinary parasites and is favorably considered for the treatment and control of helminth infections in humans [37]. Emodepside, an anthelmintic drug of the cyclooctadepsipeptide class, is used in combination with praziquantel to treat a range of gastrointestinal nematodes in dogs and cats [38–40].

We found that all 3 drugs were highly effective at inhibiting the molting of *O*. *volvulus*, even more than ivermectin or moxidectin. The IC_50_ for inhibition of L3 molting with albendazole was 7 nM [1.9 ng/ml], and the IC_90_ was 23 nM [5.8 ng/ml]. The IC_50_ for inhibition of L3 molting with oxfendazole was 34 nM [10.7 ng/ml], and the IC_90_ was 71 nM [22.4 ng/ml] (Table 1 and S1 Fig). Albendazole and oxfendazole were less effective at inhibiting the motility of L4s, both having IC_50_ >2 μM (Table 1). In previous studies, we reported that tubulin-binding drugs (flubendazole and oxfendazole) affected the motility of L4s and L5s only after repeated treatments over 14 days in culture [13,30]. Hence, both drugs might be more effective against L3s than L4s, a stage that may require prolonged treatments and further evaluation with future studies. Albendazole is used for STH treatment as a single dose of 400 mg. At this dose, it reaches a maximum plasma concentration of 24.5 ng/ml with a half-life time of 1.53 hours (AUC of 73 ng*h/ml) [41], which covers the IC_90_ for inhibition of L3 molting. In comparison, albendazole sulfoxide, an important active metabolite of albendazole, had a much improved maximum plasma concentration of 288 ng/ml with a half-life time of 8.56 hours (AUC of 3,418 ng*h/ml) than albendazole [41] (Table 1), and which covers the IC_50_ of 8 nM [2.25 ng/ml] and IC_90_ of 70 nM [19.69 ng/ml] for inhibition of L3 molting in vitro. Oxfendazole, when administered at the doses currently being tested for efficacy against trichuriasis (whipworm infection), 30 mg/kg and 15 mg/kg, achieved a maximum plasma concentration of 5,300 ± 1,690 and 6,250 ± 1,390 ng/ml, respectively, with a half-life time of approximately 9.9 hours (AUC: 78,300 ± 2,830 to 99,500 ± 2,440 ng*h/ml) (Table 1) [42], both of which cover the IC_90_ for inhibition of L3 molting. Hence, from the perspective of preventing newly established infections with *O*. *volvulus* L3 by inhibiting their molting, oxfendazole and albendazole are additional compelling candidates to consider.

Intriguingly, emodepside was the most effective drug on both L3s and L4s; it inhibited molting with an IC_50_ of 0.7 nM [0.8 ng/ml] (which is 10, 48.5, and approximately 1,000 times more potent than albendazole, oxfendazole, and moxidectin, respectively) and an IC_90_ of 2 nM [2.2 ng/ml]. Importantly, it also inhibited the motility of L4s by day 6 with an IC_50_ of 0.5 nM [0.6 ng/ml] and an IC_90_ of 78 nM [87.3 ng/ml] (Table 1 and S1 Fig), which is also more potent than the other drugs. In the ascending dose (1 to 40 mg) human clinical trial (NCT02661178), emodepside achieved a maximum plasma concentration in the range of 18.6 to 595 ng/ml, AUC of 100 to 4,112 ng*h/ml, and half-life of 1.7 to 24.6 days depending on the dose administered, and all doses were well-tolerated (Table 1) [43]. Considering that the IC_90_ for inhibition of L3 molting and L4 motility in vitro are 2 nM and 78 nM (Table 1 and S1 Fig), respectively, these values are already covered by the PK profile of the drug starting at 2.5 mg. Hence, the clinical trials for emodepside as a macrofilaricidal drug, if efficacious at 2.5 mg or above, could have additional implications in terms of utilizing emodepside for prophylactic potential.

We propose that all 5 drugs are effective against the early stages of *O*. *volvulus* based on their efficacy (IC_50_/IC_90_) in vitro. However, based on their known pharmacokinetic profiles in humans, they can be prioritized for future evaluation for their utility for prophylactic activity in humans as follows: emodepside > moxidectin > albendazole > oxfendazole > ivermectin. Moreover, we believe that the addition of some of these putative “prophylactic” drugs individually or in combination with the current MDA regimens against onchocerciasis would also align well with the integrated goals of the Expanded Special Project for Elimination of Neglected Tropical Diseases and possibly also expedite the elimination goals of one of the other 6 neglected tropical diseases amenable to MDA: the STH [44]. All 5 of these drugs are broad-spectrum anthelmintic drugs that are effective against STH infections [45–49], and thus may also benefit MDA programs aimed at controlling STH infections. The effects of MDA with ivermectin or albendazole on STHs (hookworms, *Ascaris lumbricoides*, and *Trichuris trichiura*) have already been explored in clinical studies [45,47,50] and were shown to have a significant impact on the STH infection rates in the treated communities. One dose of moxidectin (8 mg) in combination with albendazole (400 mg) was as effective as a combination of albendazole and oxantel pamoate (currently the most efficacious treatment against *T*. *trichiura*) in reducing fecal *T*. *trichiura* egg counts [46]. Notably, oxfendazole is also being tested for its effectiveness in humans against trichuriasis (NCT03435718). Additionally, emodepside was shown to not only have a strong inhibitory activity against adult STH worms in animal models with an ED_50_ of less than 1.5 mg/kg, but also against STH larval stages in vitro with IC_50_ <4 μM for L3s [49].

We could envision that a single drug, a combination of any of these 5 drugs, or just those we have prioritized (moxidectin and emodepside), when administered also for prophylaxis against the development of new *O*. *volvulus* infection, would also protect against new STH infections. Broad-spectrum chemoprophylaxis of nematode infections in humans could potentially also save on costs and time invested toward elimination of co-endemic parasites through the administration of a combination of drugs. Moreover, considering the time-consuming process of drug discovery, the heavy costs incurred, and the excessive failure rates, the prospect of repurposing commercially available drugs used for other human or veterinary diseases for the prophylaxis of *O*. *volvulus* infection is an attractive one [31,51–54]. Repurposing of drugs could also accelerate the approval timeline for new drug indications since information regarding mechanism, dosing, toxicity, and metabolism would be readily available.

In summary, our *O*. *volvulus* in vitro drug testing studies reinforce the “old” proposition of employing MDA drugs for prophylactic strategies as well, inhibiting the development of new infections with *O*. *volvulus* in the endemic regions under MDA. We report for the first time that in vitro, emodepside, moxidectin, and ivermectin have very promising inhibitory effect on both L3s and L4s, with albendazole and oxfendazole for additional consideration. Importantly, considering that the L4 larvae are longer lived as compared to the L3 stage, and hence the more feasible target against the establishment of new infections, we believe that targeting the L4 stage would be an invaluable tool toward advancing sustainable elimination goals for onchocerciasis. Moxidectin and emodepside with their superior half-life and pharmacokinetic profiles in humans and their efficacy in vitro against both L3 and L4 stages of the parasite seem to show the most promise for this purpose. Of significance, the doses required to provide exposures that would cover the IC_90_ achieved by these 2 drugs in vitro against L3 and emodepside against L4 have been shown to be well-tolerated in humans (Table 1). Crucially, as these new drugs are rolled out for human use as microfilaricidal and/or macrofilaricidal drugs, it would be important to add to the clinical protocols to also observe their effects on the development of new infections in populations that are exposed to active transmission using serological assays that can predict new infections and distinguish them from earlier infections [55]. This could potentially reveal valuable information to foster the development of more complementary elimination programs that not only target the microfilariae (moxidectin) and the adult worms (emodepside) but also the other infectious stages of the parasite, with their effects on STH being an added advantage.

Mathematical modeling has long influenced the design of intervention policies for onchocerciasis and predicted the potential outcomes of various regimens used by the elimination programs and the feasibility of elimination [56–60]. We believe that a revised mathematical model that also takes into account the additional aspect of targeting L3 and L4 stages could be helpful to assess the enhanced impact this complementary tool might have in advancing the goal of elimination, and accordingly support a revised policy for operational intervention programs first for onchocerciasis, and perhaps also as a pan-nematode control measure, by the decision-making bodies [7,61,62]. Given that in human clinical trials in which infected people were treated quarterly with ivermectin, there was an indication of a considerable trend of reduced number of newly formed nodules, it becomes apparent that the recommendation for such a revised regimen might also support protection from new infections. Clinical trials to assess the efficacy of biannual doses of ivermectin or moxidectin versus annual doses of these drugs against onchocerciasis have been already initiated (NCT03876262). Alternatively, increasing the frequency of future treatments with moxidectin and/or emodepside to biannual or quarterly treatment and/or using them in combinations could also improve their chemotherapeutic potential by targeting multiple stages of the parasite, thus increasing all the control potential of these new MDA drugs on multiple stages of the parasite and ultimately support not only a faster timeline but also sustained elimination.

## Supporting information

S1 Fig Effects of “prophylactic” drugs on *O*. *volvulus* L3 and L4 worms. The graphs show the IC_50_ and IC_90_ for inhibition of L3 molting (A–D) and inhibition of L4 motility (E–G) in the presence of ivermectin and moxidectin (A, E, and F), albendazole and oxfendazole (B), albendazole sulfoxide (C), or emodepside (D and G). The graphs are a representation of 2 separate assays, with each treatment condition tested in duplicate.
(TIFF)

S1 Text Description of the L3 molting and the inhibition of L4 motility assays. (DOCX)

## References

[1] Onchocerciasis. Keys Facts. [Internet]. 2018 [cited 2019 May 26]. Available from: https://www.who.int/news-room/fact-sheets/detail/onchocerciasis.

[2] NTD Modelling Consortium Onchocerciasis Group. The World Health Organization 2030 goals for onchocerciasis: Insights and perspectives from mathematical modelling: NTD Modelling Consortium Onchocerciasis Group. Gates Open Res. 2019;3:1545 10.12688/gatesopenres.13067.1 31723729 PMC6820451

[3] Cupp E, Sauerbrey M, Cama V, Eberhard M, Lammie PJ, Unnasch TR. Elimination of onchocerciasis in Africa by 2025: the need for a broad perspective. Infect Dis Poverty. 2019;8(1):50 10.1186/s40249-019-0557-1 31303176 PMC6628485

[4] Sauerbrey M. The Onchocerciasis Elimination Program for the Americas (OEPA). Ann Trop Med Parasitol. 2008;102(Suppl 1):25–9. 10.1179/136485908X337454 . 18718151

[5] Dadzie KY. Onchocerciasis control: the APOC strategy. Afr Health. 1997;19(3):13–5. . 12292398

[6] Rebollo MP, Zoure H, Ogoussan K, Sodahlon Y, Ottesen EA, Cantey PT. Onchocerciasis: shifting the target from control to elimination requires a new first-step-elimination mapping. Int Health. 2018;10(Suppl 1):i14–i9. Epub 2018 Feb 23. 10.1093/inthealth/ihx052 29471341 PMC5881272

[7] Walker M, Pion SDS, Fang H, Gardon J, Kamgno J, Basanez MG, et al Macrofilaricidal Efficacy of Repeated Doses of Ivermectin for the Treatment of River Blindness. Clin Infect Dis. 2017;65(12):2026–34. 10.1093/cid/cix616 29020189 PMC5850622

[8] Taylor HR. Ivermectin treatment of onchocerciasis. Aust N Z J Ophthalmol. 1989;17(4):435–8. 10.1111/j.1442-9071.1989.tb00567.x . 2696499

[9] Basáñez MG, Pion SDP, Boakes E, Filipe JAN, Churcher TS, Boussinesq M. Effect of single-dose ivermectin on *Onchocerca volvulus*: a systematic review and meta-analysis. Lancet Infect Dis. 2008;8(5):310–22. 10.1016/S1473-3099(08)70099-9 . 18471776

[10] Churcher TS, Pion SD, Osei-Atweneboana MY, Prichard RK, Awadzi K, Boussinesq M, et al Identifying sub-optimal responses to ivermectin in the treatment of River Blindness. Proc Natl Acad Sci U S A. 2009;106(39):16716–21. 10.1073/pnas.0906176106 19805362 PMC2757820

[11] Boussinesq M, Gardon J, Gardon-Wendel N, Kamgno J, Ngoumou P, Chippaux JP. Three probable cases of Loa loa encephalopathy following ivermectin treatment for onchocerciasis. Am J Trop Med Hyg. 1998;58(4):461–9. Epub 1998 May 9. 10.4269/ajtmh.1998.58.461 . 9574793

[12] Kim YE, Remme JH, Steinmann P, Stolk WA, Roungou JB, Tediosi F. Control, elimination, and eradication of river blindness: scenarios, timelines, and ivermectin treatment needs in Africa. PLoS Negl Trop Dis. 2015;9(4):e0003664 10.1371/journal.pntd.0003664 25860569 PMC4393239

[13] Hubner MP, Martin C, Specht S, Koschel M, Dubben B, Frohberger SJ, et al Oxfendazole mediates macrofilaricidal efficacy against the filarial nematode Litomosoides sigmodontis in vivo and inhibits Onchocerca spec. motility in vitro. PLoS Negl Trop Dis. 2020;14(7):e0008427 10.1371/journal.pntd.0008427 32628671 PMC7365463

[14] Hawryluk NA. Macrofilaricides: An Unmet Medical Need for Filarial Diseases. ACS Infect Dis. 2020;6(4):662–71. 10.1021/acsinfecdis.9b00469 . 32091199

[15] Geary TG, Mackenzie CD, Silber SA. Flubendazole as a macrofilaricide: History and background. PLoS Negl Trop Dis. 2019;13(1):e0006436 10.1371/journal.pntd.0006436 30650160 PMC6334891

[16] Boussinesq M. A new powerful drug to combat river blindness. Lancet. 2018;392(10154):1170–2. 10.1016/S0140-6736(18)30101-6 . 29361336

[17] Opoku NO, Bakajika DK, Kanza EM, Howard H, Mambandu GL, Nyathirombo A, et al Single dose moxidectin versus ivermectin for Onchocerca volvulus infection in Ghana, Liberia, and the Democratic Republic of the Congo: a randomised, controlled, double-blind phase 3 trial. Lancet. 2018;392(10154):1207–16. 10.1016/S0140-6736(17)32844-1 29361335 PMC6172290

[18] Milton P, Hamley JID, Walker M, Basáñez MG. Moxidectin: an oral treatment for human onchocerciasis. Expert Rev Anti Infect Ther. 2020:1–15. Epub 2020 Jul 28. 10.1080/14787210.2020.1792772 . 32715787

[19] McTier TL, Six RH, Pullins A, Chapin S, Kryda K, Mahabir SP, et al Preventive efficacy of oral moxidectin at various doses and dosage regimens against macrocyclic lactone-resistant heartworm (Dirofilaria immitis) strains in dogs. Parasit Vectors. 2019;12(1):444 10.1186/s13071-019-3685-3 31506088 PMC6737633

[20] Prichard RK, Geary TG. Perspectives on the utility of moxidectin for the control of parasitic nematodes in the face of developing anthelmintic resistance. Int J Parasitol Drugs Drug Resist. 2019;10:69–83. 10.1016/j.ijpddr.2019.06.002 31229910 PMC6593148

[21] Oxfendazole. Available from: https://www.dndi.org/diseases-projects/portfolio/oxfendazole/.

[22] Campbell WC. Lessons from the History of Ivermectin and Other Antiparasitic Agents. Annu Rev Anim Biosci. 2016;4:1–14. 10.1146/annurev-animal-021815-111209 . 26515271

[23] Taylor HR, Trpis M, Cupp EW, Brotman B, Newland HS, Soboslay PT, et al Ivermectin prophylaxis against experimental Onchocerca volvulus infection in chimpanzees. Am J Trop Med Hyg. 1988;39(1):86–90. 10.4269/ajtmh.1988.39.86 . 3400802

[24] Tchakoute VL, Bronsvoort M, Tanya V, Renz A, Trees AJ. Chemoprophylaxis of Onchocerca infections: in a controlled, prospective study ivermectin prevents calves becoming infected with O. ochengi. Parasitology. 1999;118(Pt 2):195–9. 10.1017/s0031182098003680 . 10028534

[25] Njongmeta LM, Nfon CK, Gilbert J, Makepeace BL, Tanya VN, Trees AJ. Cattle protected from onchocerciasis by ivermectin are highly susceptible to infection after drug withdrawal. Int J Parasitol. 2004;34(9):1069–74. 10.1016/j.ijpara.2004.04.011 . 15313133

[26] Campillo JT, Chesnais CB, Pion SDS, Gardon J, Kamgno J, Boussinesq M. Individuals living in an onchocerciasis focus and treated three-monthly with ivermectin develop fewer new onchocercal nodules than individuals treated annually. Parasit Vectors. 2020;13(1):258 10.1186/s13071-020-04126-x 32414398 PMC7229600

[27] Turner HC, Walker M, Attah SK, Opoku NO, Awadzi K, Kuesel AC, et al The potential impact of moxidectin on onchocerciasis elimination in Africa: an economic evaluation based on the Phase II clinical trial data. 10.1186/s13071-015-0779-4 2015;8:167. PubMed Central PMCID: PMC4381491. 25889256 PMC4381491

[28] Akama T, Freund YR, Berry PW, Carter DS, Easom EE, Jarnagin K, et al Macrofilaricidal Benzimidazole-Benzoxaborole Hybrids as an Approach to the Treatment of River Blindness: Part 1. Amide Linked Analogs. ACS Infect Dis. 2020;6(2):173–9. 10.1021/acsinfecdis.9b00396 . 31876154 PMC7026885

[29] Carter DS, Jacobs RT, Freund YR, Berry PW, Akama T, Easom EE, et al Macrofilaricidal Benzimidazole-Benzoxaborole Hybrids as an Approach to the Treatment of River Blindness: Part 2. Ketone Linked Analogs. ACS Infect Dis. 2020;6(2):180–5. 10.1021/acsinfecdis.9b00397 31876143 PMC7026882

[30] Voronin D, Tricoche N, Jawahar S, Shlossman M, Bulman CA, Fischer C, et al Development of a preliminary in vitro drug screening assay based on a newly established culturing system for pre-adult fifth-stage Onchocerca volvulus worms. PLoS Negl Trop Dis. 2019;13(1):e0007108 Epub 2019 Jan 18. 10.1371/journal.pntd.0007108 30653499 PMC6353222

[31] Bulman CA, Bidlow CM, Lustigman S, Cho-Ngwa F, Williams D, Rascon AA Jr, et al Repurposing auranofin as a lead candidate for treatment of lymphatic filariasis and onchocerciasis. PLoS Negl Trop Dis. 2015;9(2):e0003534 10.1371/journal.pntd.0003534 25700363 PMC4336141

[32] Korth-Bradley JM, Parks V, Wagner F, Chalon S, Gourley I, Matschke K, et al Effect of moxidectin on CYP3A4 activity as evaluated by oral midazolam pharmacokinetics in healthy subjects. Clin Pharmacol Drug Dev. 2014;3(2):151–7. 10.1002/cpdd.81 . 27128460

[33] Baraka OZ, Mahmoud BM, Marschke CK, Geary TG, Homeida MM, Williams JF. Ivermectin distribution in the plasma and tissues of patients infected with Onchocerca volvulus. Eur J Clin Pharmacol. 1996;50(5):407–10. 10.1007/s002280050131 . 8839664

[34] Awadzi K, Opoku NO, Attah SK, Lazdins-Helds J, Kuesel AC. A randomized, single-ascending-dose, ivermectin-controlled, double-blind study of moxidectin in Onchocerca volvulus infection. PLoS Negl Trop Dis. 2014;8(6):e2953 10.1371/journal.pntd.0002953 24968000 PMC4072596

[35] Savioli L, Albonico M, Daumerie D, Lo NC, Stothard JR, Asaolu S, et al Review of the 2017 WHO Guideline: Preventive chemotherapy to control soil-transmitted helminth infections in at-risk population groups. An opportunity lost in translation. PLoS Negl Trop Dis. 2018;12(4):e0006296 10.1371/journal.pntd.0006296 29698486 PMC5919405

[36] Ichimori K, King JD, Engels D, Yajima A, Mikhailov A, Lammie P, et al Global programme to eliminate lymphatic filariasis: the processes underlying programme success. PLoS Negl Trop Dis. 2014;8(12):e3328 10.1371/journal.pntd.0003328 25502758 PMC4263400

[37] Gonzalez AE, Codd EE, Horton J, Garcia HH, Gilman RH. Oxfendazole: a promising agent for the treatment and control of helminth infections in humans. Expert Rev Anti Infect Ther. 2019;17(1):51–6. 10.1080/14787210.2018.1555241 30501436 PMC6376865

[38] Altreuther G, Radeloff I, LeSueur C, Schimmel A, Krieger KJ. Field evaluation of the efficacy and safety of emodepside plus praziquantel tablets (Profender tablets for dogs) against naturally acquired nematode and cestode infections in dogs. Parasitol Res. 2009;105(Suppl 1):S23–9. 10.1007/s00436-009-1492-z . 19575222

[39] Schimmel A, Altreuther G, Schroeder I, Charles S, Cruthers L, Kok DJ, et al Efficacy of emodepside plus praziquantel tablets (Profender tablets for dogs) against mature and immature adult Trichuris vulpis infections in dogs. Parasitol Res. 2009;105(Suppl 1):S17–22. 10.1007/s00436-009-1491-0 . 19575221

[40] Bohm C, Wolken S, Schnyder M, Basso W, Deplazes P, Di Cesare A, et al Efficacy of Emodepside/Praziquantel Spot-on (Profender(R)) against adult Aelurostrongylus abstrusus Nematodes in Experimentally Infected Cats. Parasitol Res. 2015;114(Suppl 1):S155–64. 10.1007/s00436-015-4521-0 . 26152416

[41] Schulz JD, Neodo A, Coulibaly JT, Keiser J. Pharmacokinetics of Albendazole, Albendazole Sulfoxide, and Albendazole Sulfone Determined from Plasma, Blood, Dried-Blood Spots, and Mitra Samples of Hookworm-Infected Adolescents. Antimicrob Agents Chemother. 2019;63(4). 10.1128/AAC.02489-18 30745388 PMC6437472

[42] An G, Murry DJ, Gajurel K, Bach T, Deye G, Stebounova LV, et al Pharmacokinetics, Safety, and Tolerability of Oxfendazole in Healthy Volunteers: a Randomized, Placebo-Controlled First-in-Human Single-Dose Escalation Study. Antimicrob Agents Chemother. 2019;63(4). 10.1128/AAC.02255-18 30745383 PMC6437481

[43] ClinicalTrials.gov. Available from: https://clinicaltrials.gov/ct2/show/results/NCT02661178.

[44] Hopkins AD. Neglected tropical diseases in Africa: a new paradigm. Int Health. 2016;8(Suppl 1):i28–33. Epub 2016 Mar 5. 10.1093/inthealth/ihv077 . 26940307

[45] Moncayo AL, Vaca M, Amorim L, Rodriguez A, Erazo S, Oviedo G, et al Impact of long-term treatment with ivermectin on the prevalence and intensity of soil-transmitted helminth infections. PLoS Negl Trop Dis. 2008;2(9):e293 10.1371/journal.pntd.0000293 18820741 PMC2553482

[46] Barda B, Ame SM, Ali SM, Albonico M, Puchkov M, Huwyler J, et al Efficacy and tolerability of moxidectin alone and in co-administration with albendazole and tribendimidine versus albendazole plus oxantel pamoate against Trichuris trichiura infections: a randomised, non-inferiority, single-blind trial. Lancet Infect Dis. 2018;18(8):864–73. 10.1016/S1473-3099(18)30233-0 . 29858149

[47] Pion SDS, Chesnais CB, Awaca-Uvon NP, Vlaminck J, Abdou A, Kunyu-Shako B, et al The impact of four years of semiannual treatments with albendazole alone on lymphatic filariasis and soil-transmitted helminth infections: A community-based study in the Democratic Republic of the Congo. PLoS Negl Trop Dis. 2020;14(6):e0008322 10.1371/journal.pntd.0008322 . 32574160 PMC7337406

[48] Vercruysse J, Behnke JM, Albonico M, Ame SM, Angebault C, Bethony JM, et al Assessment of the anthelmintic efficacy of albendazole in school children in seven countries where soil-transmitted helminths are endemic. PLoS Negl Trop Dis. 2011;5(3):e948 10.1371/journal.pntd.0000948 21468309 PMC3066140

[49] Karpstein T, Pasche V, Haberli C, Scandale I, Neodo A, Keiser J. Evaluation of emodepside in laboratory models of human intestinal nematode and schistosome infections. Parasit Vectors. 2019;12(1):226 Epub 2019 May 16. 10.1186/s13071-019-3476-x 31088525 PMC6515646

[50] Pion SDS, Chesnais CB, Weil GJ, Fischer PU, Missamou F, Boussinesq M. Effect of 3 years of biannual mass drug administration with albendazole on lymphatic filariasis and soil-transmitted helminth infections: a community-based study in Republic of the Congo. Lancet Infect Dis. 2017;17(7):763–9. 10.1016/S1473-3099(17)30175-5 . 28372977

[51] Hernandez HW, Soeung M, Zorn KM, Ashoura N, Mottin M, Andrade CH, et al High Throughput and Computational Repurposing for Neglected Diseases. Pharm Res. 2018;36(2):27 10.1007/s11095-018-2558-3 30560386 PMC6792295

[52] Johnston KL, Ford L, Umareddy I, Townson S, Specht S, Pfarr K, et al Repurposing of approved drugs from the human pharmacopoeia to target Wolbachia endosymbionts of onchocerciasis and lymphatic filariasis. Int J Parasitol Drugs Drug Resist. 2014;4(3):278–86. 10.1016/j.ijpddr.2014.09.001 25516838 PMC4266796

[53] Panic G, Duthaler U, Speich B, Keiser J. Repurposing drugs for the treatment and control of helminth infections. Int J Parasitol Drugs Drug Resist. 2014;4(3):185–200. 10.1016/j.ijpddr.2014.07.002 25516827 PMC4266803

[54] Kuesel AC. Research for new drugs for elimination of onchocerciasis in Africa. Int J Parasitol Drugs Drug Resist. 2016;6(3):272–86. 10.1016/j.ijpddr.2016.04.002 27693536 PMC5196484

[55] Bennuru S, Oduro-Boateng G, Osigwe C, Del Valle P, Golden A, Ogawa GM, et al Integrating Multiple Biomarkers to Increase Sensitivity for the Detection of Onchocerca volvulus Infection. J Infect Dis. 2020;221(11):1805–15. 10.1093/infdis/jiz307 31201416 PMC7213562

[56] Basanez MG, Walker M, Turner HC, Coffeng LE, de Vlas SJ, Stolk WA. River Blindness: Mathematical Models for Control and Elimination. Adv Parasitol. 2016;94:247–341. 10.1016/bs.apar.2016.08.003 . 27756456

[57] Verver S, Walker M, Kim YE, Fobi G, Tekle AH, Zoure HGM, et al How Can Onchocerciasis Elimination in Africa Be Accelerated? Modeling the Impact of Increased Ivermectin Treatment Frequency and Complementary Vector Control. Clin Infect Dis. 2018;66(Suppl 4):S267–S74. 10.1093/cid/cix1137 29860291 PMC5982715

[58] Stolk WA, Walker M, Coffeng LE, Basanez MG, de Vlas SJ. Required duration of mass ivermectin treatment for onchocerciasis elimination in Africa: a comparative modelling analysis. Parasit Vectors. 2015;8:552 10.1186/s13071-015-1159-9 26489937 PMC4618738

[59] Turner HC, Walker M, Lustigman S, Taylor DW, Basanez MG. Human Onchocerciasis: Modelling the Potential Long-term Consequences of a Vaccination Programme. PLoS Negl Trop Dis. 2015;9(7):e0003938 10.1371/journal.pntd.0003938 26186715 PMC4506122

[60] Coffeng LE, Stolk WA, Hoerauf A, Habbema D, Bakker R, Hopkins AD, et al Elimination of African onchocerciasis: modeling the impact of increasing the frequency of ivermectin mass treatment. PLoS One. 2014;9(12):e115886 10.1371/journal.pone.0115886 25545677 PMC4278850

[61] Walker M, Hamley JID, Milton P, Monnot F, Pedrique B, Basanez MG. Designing antifilarial drug trials using clinical trial simulators. Nat Commun. 2020;11(1):2685 10.1038/s41467-020-16442-y 32483209 PMC7264235

[62] Behrend MR, Basanez MG, Hamley JID, Porco TC, Stolk WA, Walker M, et al Modelling for policy: The five principles of the Neglected Tropical Diseases Modelling Consortium. PLoS Negl Trop Dis. 2020;14(4):e0008033 10.1371/journal.pntd.0008033 32271755 PMC7144973

