# Supplementary figures and images for: Drugs that target early stages of Onchocerca volvulus: A revisited means to facilitate the elimination goals for onchocerciasis
Source: PLoS Negl Trop Dis. 2021 Feb 18;15(2):e0009064. doi: 10.1371/journal.pntd.0009064 (PMC7891776; doi:10.1371/journal.pntd.0009064)

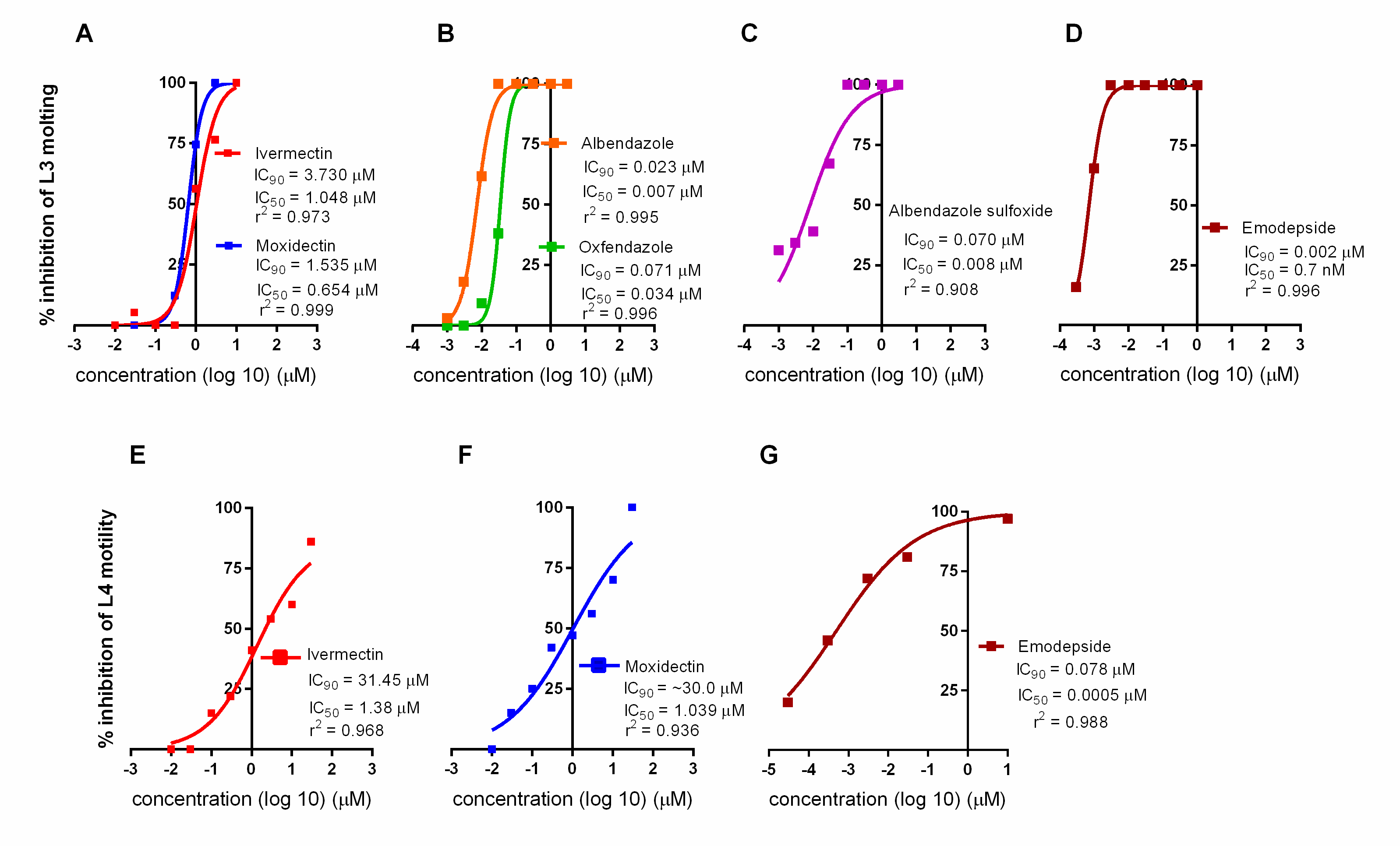

Supplement: S1 Fig — The graphs show the IC50 and IC90 for inhibition of L3 molting (A–D) and inhibition of L4 motility (E–G) in the presence of ivermectin and moxidectin (A, E, and F), albendazole and oxfendazole (B), albendazole sulfoxide (C), or emodepside (D and G). The graphs are a representation of 2 separate assays, with each treatment condition tested in duplicate. (TIFF) [file pntd.0009064.s001.tiff]
