# Supplementary material for: Drugs that target early stages of Onchocerca volvulus: A revisited means to facilitate the elimination goals for onchocerciasis
Source: PLoS Negl Trop Dis. 2021 Feb 18;15(2):e0009064. doi: 10.1371/journal.pntd.0009064 (PMC7891776; doi:10.1371/journal.pntd.0009064)
Supplement: S1 Text — (DOCX) [file pntd.0009064.s002.docx]

**Supplemental Methods:**

**Inhibition of L3 molting:**

Cryopreserved *O*. *volvulus* L3 stage larvae were thawed and washed in medium that contains 1:1 NCTC-109 and IMDM supplemented with Glutamax (1x) and 2x Antibiotic-Antimycotic (Life Technologies). After washing, the larvae were dispersed in a 96-well plate (10 larvae/well) containing 1.5x10^5^ human Peripheral Mononuclear Blood Cells (PBMC) per well in 100 µl complete medium, which was supplemented with heat inactivated 20% fetal bovine serum (FBS, Sigma). Worms were cultured with and without various concentration of the drug at 37 ˚C in a 5% CO_2_ incubator until day 6 when the molting rate was estimated under an inverted microscope based on the presence of the highly motile L4 and the empty L3 cuticles. The percent inhibition of molting was calculated based on the number of treated larvae that were able to molt in comparison to the number of control larvae that had successfully molted.

Tested compounds were dissolved in DMSO at a concentration of 10 mM and added to the L3s in 100 µl of complete media containing 2x the desired final concentration. Ivermectin (PHR1380, Sigma Aldrich Inc.) and moxidectin (PHR1827, Sigma Aldrich Inc.) were tested in the range of 0.01 - 10 µM, albendazole (A4673, Sigma Aldrich), albendazole sulfoxide (35395, Sigma Aldrich) and oxfendazole (31476, Sigma Aldrich Inc.) in the range of 1 nM - 3 µM, and emodepside (Bayer) in the range of 0.3 nM – 1 µM using 3-fold dilutions. Each condition was tested in duplicates and repeated at least once. The IC_50_ and IC_90_ were derived from non-linear regression (curve fit) analysis on GraphPad Prism 6 with 95% confidence intervals.

**Inhibition of L4 motility:**

L3 washed larvae were dispersed in a 96-well plate (10 larvae/well) containing 1.5 x10^5^ PBMC per well in complete medium at 37 ˚C and in 5% CO_2_ incubator until day 5. The highly motile L4s were then transferred into a petri dish containing complete medium and aliquots of 6 - 8 worms were retrieved from the petri dish and placed into a 96-well plate (8-10 larvae/well) in 100 µl of media. Tested compounds were dissolved in DMSO at a concentration of 10 mM and added to the L4s in 100 µl of complete media containing 2x the desired final concentration. Ivermectin and moxidectin: 0.01 to 30 µM at 3-fold dilutions, and emodepside: 0.03 nM to 3 µM at 10-fold dilutions and 10 µM. Motility was evaluated manually every day under an inverted microscope until day 6. Each condition was tested in duplicates and repeated at least once. Motility was scored based on the following scale: 100% motility, constant coiling movement; 75% motility, slower coiling; 50% motility, slow and intermittent movement; 25% motility, very slow movement, or twitching; and 0% motility, no movement. The percent inhibition of motility was calculated based on the % motility of the treated larvae in comparison to % motility of control larvae. The IC_50_ and IC_90_ were derived from non-linear regression (curve fit) analysis on GraphPad Prism 6 with 95% confidence intervals.
